# Targeting Aromatase and 5-α-Reductase to Limit Dysfunctional Adipogenesis in Multiple Symmetric Lipomatosis

**DOI:** 10.3390/biomedicines14081706

**Published:** 2026-07-29

**Authors:** Gabriella Milan, Chiara Compagnin, Isabel Zucal, Giuseppe Perale, Silvia Bettini, Anna Pilatone, Marco De Monti, Yves Harder, Corrado Parodi, Daniel Schmauss, Vincenzo Vindigni, Franco Bassetto, Oliver Felthaus, Dmytro Oliinyk, Lukas Prantl, Luca Busetto

**Affiliations:** 1 Internal Medicine 3, Endocrine-Metabolic Laboratory, Department of Medicine, University of Padova, Padova University Hospital, Via Ospedale 105, 35128 Padova, Italy; 2 Center for the Study and the Integrated Treatment of Obesity, Padova University Hospital, 35128 Padova, Italy; 3 Department of Plastic, Reconstructive and Aesthetic Surgery and Hand Surgery, Centre Hospitalier Universitaire Vaudois (CHUV), 1011 Lausanne, Switzerlandyves.harder@chuv.ch (Y.H.); 4 Faculty of Biology and Medicine, University of Lausanne (UNIL), 1015 Lausanne, Switzerland; 5 Department of Plastic, Reconstructive and Aesthetic Surgery, Ospedale Regionale di Lugano, Ente Ospedaliero Cantonale (EOC), 6900 Lugano, Switzerlanddaniel.schmauss@eoc.ch (D.S.); 6 Faculty of Biomedical Sciences, University of Southern Switzerland (USI), 6900 Lugano, Switzerlandmarco.demonti@eoc.ch (M.D.M.); 7 Ludwig Boltzmann Institute for Experimental and Clinical Traumatology, 1200 Vienna, Austria; 8 Department of General Surgery, Ospedale Regionale di Mendrisio, Ente Ospedaliero Cantonale (EOC), 6850 Mendrisio, Switzerland; 9 Plastic Surgery Unit, Department of Neurosciences, University of Padova, 35128 Padova, Italy; vincenzo.vindigni@unipd.it (V.V.);; 10 Department for Plastic, Hand & Reconstructive Surgery, University Hospital Regensburg, 93053 Regensburg, Germanylukas.prantl@klinik.uni-regensburg.de (L.P.); 11 Clinical Nutrition Unit, Department of Medicine, University of Padova, Padova University Hospital, 35128 Padova, Italy

**Keywords:** multiple symmetric lipomatosis, Madelung’s disease, orphan disease, subcutaneous adipose tissue, lipomatous tissue, 5-α-reductase inhibitor, aromatase inhibitor, DL-α-Lipoic Acid

## Abstract

**Background**: Multiple symmetric lipomatosis (MSL) is an orphan disease characterized by pathological expansion of lipomatous tissue (LT) in the subcutaneous adipose tissue (SAT) areas, causing severe deformities and compression of vital organs. To date, the only treatment options available are either lifestyle modification or surgical excision/liposuction. Since sex hormones play an important role in adipose tissue (AT) expansion, they may represent an opportunity for pharmacological treatment. **Methods**: LT and control SAT were collected from eight MSL patients. Both tissues and derived stromal vascular fraction (SVF) were compared in terms of aromatase (*hCYP19A1*) and 5-α-reductase isoform (*hSRD5A1*, *-2* and *-3*) mRNA expression. Primary cultures obtained from one MSL patient were exposed to an aromatase inhibitor, 5-α-reductase inhibitor and DL-α-Lipoic Acid and assayed for cell viability, proliferation and adipogenic differentiation. **Results**: *hCYP19A1*, *hSRD5A1* and *hSRD5A3* were expressed in both MSL-affected and MSL-spared tissues at different levels. Cell viability and proliferation of LT-derived SVF cells were slightly increased by treatment with DL-α-Lipoic Acid and the aromatase inhibitor. Adipogenesis was slightly reduced by Finasteride and strongly reduced by Exemestane (more in LT- than in SAT- derived primary cultures). **Conclusions**: These preliminary results pave the way for the use of aromatase and 5-α-reductase inhibition as a potential pharmacological approach to tackling dysfunctional adipogenesis in MSL. Therefore, the reported study should be interpreted as exploratory and further experiments are recommended in a larger number of patients to validate these promising results.

## 1. Introduction

Multiple symmetric lipomatosis (MSL), also known as Madelung’s disease, is a rare condition [1,2,3,4] with an estimated incidence of 1:25,000 [2,4]. This disease is characterized by the symmetrical accumulation of non-encapsulated fatty masses, originating the lipomatous tissue (LT), which localizes primarily around the neck, shoulder girdle, upper arms and thorax. According to the classification of Schiltz et al. [2], MSL is divided into three main subtypes based on fat accumulation areas. Specifically, type I MSL is characterized by fat depots mainly on the neck, shoulder girdle, upper arms and trunk. Type II MSL is marked by fat depots on the hips, bottom and upper legs while type III MSL shows fat depots throughout the entire body, excluding the head, forearms and lower legs. LT-masses are mobile and can cause severe deformities and morbidity due to their size and location. The condition can lead to compression of vital organs through intra-mediastinal and intra-abdominal growth, making it chronically debilitating and potentially life-threatening [5].

The current treatment options include lifestyle modifications and surgery [6], often combined. Regarding lifestyle modifications, abstinence from alcohol as well as healthy diet and exercise are advised to avoid further LT growth. On the other hand, surgery refers to surgical excision or liposuction of LT-masses [2,6]. Despite surgical treatment, which cannot be indefinitely repeated, no pharmaceutical treatment has been proposed yet. In this regard, topical treatment seems of particular interest, since LT is easily accessible.

Earlier studies describe LT adipose cells as smaller in volume, suggesting growth by hyperplastic rather than hypertrophic mechanisms, and identify impaired lipolysis as a contributing factor in MSL development [7]. Current research suggests the role of stem cells and their (hyper-)proliferation as one of the possible causes [8].

The origin of LT and the morphological and functional characteristics of this peculiar tissue deposit are not completely understood, although some authors have suggested a relationship with brown adipose tissue (BAT) [9]. In particular, no significant differences have been observed in β-adrenoceptor subtype mRNA levels between adipocytes from MSL patients and healthy controls. Additionally, no functional mutations have been identified in the β3-adrenoceptor, the subtype primarily responsible for modulating thermogenic gene expression in BAT. Notably, genes upregulated by noradrenaline—such as mitochondrial uncoupling protein 1 (UCP1), inducible nitric oxide synthase (iNOS), and peroxisome proliferator-activated receptor γ co-activator 1 (PGC-1)—are expressed in MSL-derived adipocytes but are not inducible by noradrenaline stimulation [10]. These findings suggest a functional uncoupling of adrenergic signaling and highlight the need for alternative pathways, such as local steroid metabolism, to be explored as therapeutic targets.

We recently reported, in a cohort of six MSL type I patients, that LT displayed white subcutaneous adipose tissue (SAT) morphology and expression patterns, without increased levels of the brown-specific marker UCP1 or augmented browning capacity. However, a distinct AKT/Protein Kinase B (PKB), Protein Kinase CK2 and Extracellular Signal-Regulated Kinase (ERK1/2) hyperactivation has been demonstrated in LT. Moreover, the stromal vascular fraction (SVF) from LT contained an increased number of adipose stem cells (ASCs) that proliferated faster, formed clones, and differentiated into white adipocytes more efficiently than those of the control. Thus, this data suggested that LT appears as a white SAT depot expanding by hyperplasia through increased stemness and enhanced white adipogenesis, upregulating different specific kinases and related pathways, which could represent new therapeutic targets [11].

Sex hormones are critical regulators of AT growth and metabolism in humans [12]. Specifically, sex steroid hormones have been demonstrated to modulate visceral versus subcutaneous lipid accumulation that correlates with abdominal obesity and metabolic syndrome [13]. Moreover, AT is also considered a direct source of sex hormones with paracrine regulatory effects on SAT itself [12]. In particular, aromatase (CYP19A1), a member of the cytochrome P450 superfamily, catalyzes the conversion of androgens to estrogens, a rate-limiting step in estrogen biosynthesis [14,15]. Aromatase is expressed in different cell types and tissues including ovarian cells, testicular Sertoli and Leydig cells, placental tissue [16], SAT [17,18], skin fibroblasts and different regions of the brain [19]. Regarding SAT, a recent study observed that aromatase levels were higher in SAT from males with obesity compared to those without obesity, and *CYP19A1* expression correlated positively with adiposity, hyperglycemia, and insulin resistance [20]. The steroid 5-α-reductase (SRD5A) enzyme family comprises three isoforms (SRD5A1, -2 and -3) that produce steroid hormones which primarily regulate male sexual development. SRD5A2 is the major 5-α-reductase isoenzyme in the prostate. SRD5A3 has been linked to N-linked glycosylation of proteins [21,22] and hormone-refractory prostate cancer [23], and can be inhibited by both Dutasteride and Finasteride [24]. Among all tissues analyzed by Yamana et al. [24]—including the normal prostate, mammary gland, brain, skin and AT—SRD5A3 is more highly expressed than SRD5A1 and SRD5A2. However, in whole human AT, both in visceral (omental) and subcutaneous depots, SRD5A3 was the most highly expressed isoenzyme followed by SRD5A1; conversely, the SRD5A2 was not expressed [25].

LT growth in MSL could potentially be modulated by targeting local intracellular sex hormone synthesis, activity, and metabolism. Recent evidence suggests that inactivation of the peripheral aromatase activity in the fat cells and fibroblasts reduces local estradiol concentration and therefore adipogenesis [26]. This would also lead to an increase in intracellular testosterone levels and consequently stimulate lipolysis. The local parallel inhibition of the 5-α-reductase activity could further increase the intracellular testosterone levels and promote local fat loss, as reported in hypogonadal patients with obesity [27]. Lastly, the antioxidant and histone deacetylase inhibitor DL-α-Lipoic Acid (DL-α-LA) has been shown to have a synergistic effect with the inhibitor of aromatase activity in estrogen receptor-positive breast cancer cells [28].

In the present study, we aimed to analyze the expression and activity of these enzymes in the LT of MSL patients and to test the effects of specific chemical inhibitors—Finasteride (SRD5A inhibitor) and Exemestane (CYP19A1 inhibitor)—as well as DL-α-Lipoic Acid on cell viability, proliferation and adipogenesis of primary cell cultures derived from LT-SVF. In this preclinical setting, the goal is to investigate the feasibility and the efficacy of this new potential treatment to tackle dysfunctional adipogenesis in MSL.

## 2. Materials and Methods

### 2.1. Reagent and Stock Solutions

1β-^3^H(N)-Androst-4-ene-3,17-dione (Perkin Elmer, Waltham, MA, USA) 250 µCi (9.25 MBq), specific activity 25.6 Ci/mmol (0.947TBq/mmol) in 250 µL of ethanol; DL-α-Lipoic Acid (LA) (Cayman Chemical, Ann Arbor, MI, USA), 121 mM in ethanol (484×); Finasteride (F) (Cayman Chemical), 67 mM in ethanol (1340×); Exemestane (E) (Cayman Chemical), 84 mM in DMSO (1680×); Transferrin (Sigma-Aldrich, Merck KGaA, Darmstadt, Germany) 10 mg/mL (1000×); D-Biotin (Sigma-Aldrich, Biomedicals, Santa Ana, CA, USA) 33 mM in NaOH 1N (1000×); D-Pantothenic acid calcium salt (MP Biomedicals, Santa Ana, CA, USA) 170 mM in culture medium (10,000×); Dexamethasone (Sigma-Aldrich) 2.5 mM in ethanol (25,000×); T3 (3,3′,5Triiodio-L-Thyronine) (Sigma-Aldrich) 2 mg/mL in NaOH 1N (3.07 × 10^6^×); Insulin, Humulin R (Eli Lilly, Indianapolis, IN, USA) 100 UI/mL (600 µM) (8751×); IBMX (3-isobutyl-1-methylxanthine) (Sigma-Aldrich) 500 mM in DMSO (2000×); Rosiglitazone (AdooQ BioScience, Irvine, CA, USA) 10 mM in DMSO (1000×).

### 2.2. Cell Lines

JEG3 human placenta-derived cell line (ATCC number HTB-36) was cultured in MEM 10%FBS (GIBCO, Thermo Fisher Scientific, Waltham, MA, USA). 3T3-L1 mouse preadipocyte cell line (ATCC number CL-173) was cultured in DMEM high-glucose 10%FBS (GIBCO).

### 2.3. Patient Characterization and Tissue Collection for RNA Expression Experiments

Six patients (ID 2884, 2891, 3002, 3030, 5091, 5937) affected by type 1 MSL, which has been previously described [9], and two patients (ID 3662, 4450) affected by type 2 MSL who underwent lipectomy for clinical indication at the Institute of Plastic and Reconstructive Surgery of Padua Hospital were enrolled after providing written informed consent (10/4/2014 Prot. N° 2658P approved by the Padua Ethical Committee for Clinical Research). LT and a paired control SAT biopsy in MSL-spared site were collected from the same patient during surgical lipectomy. In two patients (2891 and 3002), samples of both LT and SAT were collected twice during two distinct surgeries, indicated as #1 and #2. LT samples were obtained from neck/cervical region (Madelung collar) (patients 2884, 2891#1 and #2, 3002#1, 5091) and from upper arms (patients 3002#1, 3030, 3662, 4450, 5091, 5937), whereas control SAT was obtained from the abdominal or lumbar region (Table 1). Patient age ranged from 49 to 70 years, and at least one year of alcohol abstinence was requested as inclusion criterion for enrollment into the study. For each patient, at the time of each surgery, anthropometric parameters (weight, height) were collected and body mass index (BMI) was computed [weight in kilograms/(height in meters)^2^]; photographic documentation was performed in order to assess the sites and extension of the disease. The high prevalence of type I MSL in males is reflected in our cohort, in which there are only two females out of six patients. Three patients were affected by Type 2 Diabetes Mellitus (T2DM) and seven patients had a history of alcohol abuse. BMI ranged from 22.5 to 36.7 kg/m^2^. Clinical data and the sites of LT and SAT collection are reported in Table 1.

### 2.4. Patient Characterization and Tissue Collection for Primary Cell Culture Experiments

One MSL patient (male, 57 years old, ID 20922) with a clinical presentation combining MSL types 1–3 [ethical committee number 24-3802-101, University of Regensburg, Germany] underwent lipectomy at the University of Regensburg (Germany). Biopsies of LT from the affected right thigh medially and control SAT from the non-affected right calf medially were collected and immediately transferred to standard culture medium at 4 °C. LT contained more vessels and fibrous tissue than the control SAT.

### 2.5. Isolation of Stromal Vascular Fraction

After sampling, the tissues were partly frozen in liquid nitrogen and stored at −80 °C prior to RNA extraction.

When tissue availability allowed, SVF cells from LT and paired control SAT samples were freshly isolated ex vivo. Briefly, LT and SAT biopsies were minced and digested in collagenase type II solution (1 mg/mL) (Sigma-Aldrich) for 1 h at 37 °C under gentle agitation. Then biopsies were centrifuged (350× *g*, 10 min) and the red blood cells were removed by treating samples with standard lysis buffer for 5 min. At this point, freshly isolated SVF cells were counted using the Trypan Blue assay (1:2 dilution) with a Neubauer chamber, distinguishing viable (unstained) from non-viable (blue-stained) cells. Subsequently, the cells were lysed using RLT buffer (QIAGEN, Germantown, MD, USA) and frozen for RNA extraction or they were used for cell culture experiments and to test adipogenic differentiation.

### 2.6. Extraction of RNA and Real-Time PCR

Total RNA was extracted from LT and SAT biopsies (50–100 mg) and derived SVF cells, using respectively RNeasy Lipid or Mini Kits (QIAGEN) following the supplier’s instructions. RNA content was quantified using DS-11 Series Spectrophotometer/Fluorometer technology (DeNovix, Wilmington, DE, USA). A total of 200 ng of RNA were treated with DNase Treatment and Removal Reagents (Thermo Fisher Scientific, Waltham, MA, USA) and reverse-transcribed for 1 h at 37 °C with 150 ng random primers, 0.5 mM dNTPs, 20 units of RNAsin Ribonuclease Inhibitor, and 200 units of M-MLV RT (Promega Corporation, Madison, WI, USA). Real-Time PCR was carried out with Platinum^®^ SYBR^®^ Green qPCR SuperMix-UDG (Thermo Fisher Scientific) on a DNA Engine Opticon^TM^ 2 Continuous Fluorescence Detection System (MJ Research, Waltham, MA, USA). Primer sequences and amplification conditions are shown in Table 2. The expression of each mRNA was measured in duplicate 5 ng cDNA samples, normalized by RNA18S content, and reported in arbitrary units (AUs). For *CYP19A1* the standard curve was performed using cDNA dilution series obtained from placenta tissue that serves as positive control. For *SRD5A* the standard curve was performed using cDNA dilution series obtained from primary fibroblast cultures (Figure S1).

### 2.7. Quantification of Aromatase Enzyme Activity

Aromatase enzyme activity was measured using a tritiated water-release assay as described by Lephart and Simpson [29] with minor modification for in vitro cell cultures [30]. Cells were seeded in 12-well plates in the specific medium in replicates at day 0 and treated with the different inhibitors the day after (day 1) for 4 h. After washing with PBS at room temperature, 250 µL of DMEM serum-free (*w*/*o* phenol red) with 150 nM 1β-^3^H(N)-Androst-4-ene-3,17-dione (1 µL/well) were added to each well for 1.5 h at 37 °C in cell incubator (5% CO_2_), in the presence of inhibitors as indicated; the reaction was stopped on ice. A total of 200 µL of medium from each well was transferred to a 1.5 mL Eppendorf tube, mixed by vortex with 500 µL of cold chloroform, and centrifuged at 12,000 *g* (rcf) for 5 min at 4 °C. The upper phase (100 µL) was mixed by vortex with 100 µL of charcoal-dextran coated (Sigma) in NaCl 0.9 solution (50 mg/mL) in 1.5 mL Eppendorf tubes, incubated for 5 min at room temperature and centrifuged at 12,000 *g* (rcf) × 15 min at 4 °C. β radioactivity (count time: 3 min) was measured with a liquid scintillation analyzer (Tri-Carb 2900TR, Perkin Elmer) using 100 µL of each sample, collected without disturbing the black pellet, upon addition of a liquid scintillation cocktail (Ultima Gold, Perkin Elmer).

### 2.8. Assay of Cell Viability and Analysis of Cell Proliferation

A total of 4 × 10^6^ SVF cells obtained from LT and control SAT of the MSL patient ID 20922 were plated in a T75 cell culture flask in DMEM F12 10%FBS (Thermo Fisher Scientific, Waltham, MA, USA) or in αMEM 5% human Platelet Lysate (hPL) (Sigma-Aldrich, St. Louis, MO, USA) for expansion. SVF cell subcultures (passage 2) were seeded 5000/well in a 96-well plate (PerkinElmer View Plate 96 for luminescence assay) in DMEM F12 10%FBS. JEG3, a human placenta choriocarcinomas-derived tumor cell line, was used as positive control, cultured and seeded (5000 cells/well) in MEM 10%FBS (GIBCO, Thermo Fisher Scientific, Waltham, MA, USA). Cells were seeded in 100 µL of indicated medium. Treatments were performed by adding 50 µL of 3X compound solutions to obtain the following final concentration: DL-α-Lipoic Acid (LA) (250 µM), Finasteride (F) (50 µM), Exemestane (E) (50 µM) and a combination of all 3 (LA + F + E). The concentration of compounds was selected in preliminary experiments based on previous reports. Cell viability was assessed using a RealTime-Glo MT Cell viability assay (G9711, Promega Corporation), which is based on monitoring the reducing potential of cells as a metabolic marker of viability. The endpoint method was used with reagent addition at the end of the test after 48 h of treatment with the different compounds, alone or in combination. The luminescence signal was detected by Victor 3 Multilabel Plate Reader (PerkinElmer, Waltham, MA, USA).

### 2.9. Adipogenic Differentiation

A total of 10,000 or 25,000 SVF cells obtained from control SAT and LT of MSL patient ID 20922 were seeded in 96-well flat-bottom plates (Falcon Becton Dickinson, Franklin Lakes, NS, USA) in 100 µL of DMEM F12 10%FBS or αMEM 5% hPL and grown until 2 days post-confluence. Cells were induced to differentiate into mature adipocytes in hMIR for 3 days and then maintained in hMAD, changing the medium every 3 days. Throughout the entire differentiation process (15–20 days), cells were treated with DL-α-Lipoic Acid (LA) (250 µM), Finasteride (F) (50 µM) and Exemestane (E) (50 µM), alone or in combination (LA + F + E). Untreated cells following the same induction and differentiation protocol served as internal control.

- hMAD (human Medium for Adipogenesis): DMEM F12 serum-free (Transferrin 10 µg/mL; D-Biotin 33 µM; D-Pantothenic acid calcium salt 17 µM); Dexamethasone 100 nM; T3 (3,3′,5Triiodio-L-Thyronine) 1 nM; Insulin 70 nM.
- hMIR (human Medium for Adipogenesis with IBMX and Rosiglitazone): hMAD with IBMX 0.25 mM and Rosiglitazone 10 µM.

At the end of the adipogenic differentiation (day 15–20) representative images of in vitro differentiated cells were acquired with a Leica DM IL LED inverted microscope. The percentage of adipogenesis was estimated and quantified by optical microscopy analysis of phase contrast images, performed by two independent qualified operators.

### 2.10. Statistical Analysis

Results are presented as mean ± SEM. Variables were tested for normality using a Shapiro–Wilk test. Statistical analyses were performed by Mann–Whitney and Multiple Mann–Whitney tests; one-way ANOVA followed by Dunnett’s post hoc test was used for multiple comparisons against the control group and two-way ANOVA with Tukey’s multiple comparisons test. All the *p* values were two-sided, and differences were considered statistically significant at a *p* value lower than 0.05. Statistical analysis was performed using GraphPad Prism version 10.6.0 software (GraphPad Software, LLC, Boston, MA, USA).

## 3. Results

### 3.1. Aromatase and 5-α-Reductase Gene Expression in MSL Patients

**Figure 1:**
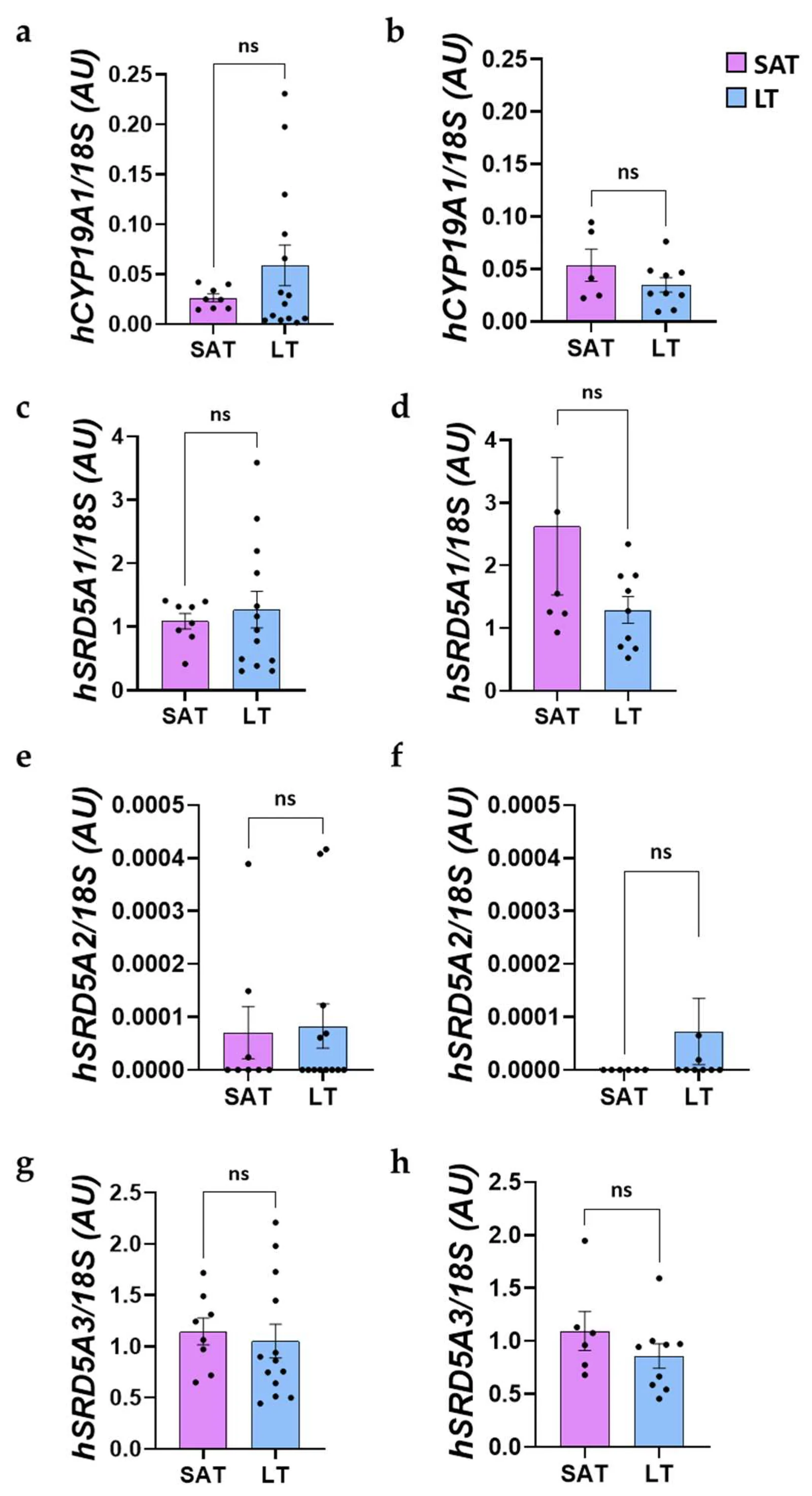
Expression profiles of *hCYP19A1*, *hSRD5A1*, *hSRD5A2* and *hSRD5A3* in whole adipose tissue and in SVF cells obtained from 8 MSL patients. (**a**,**b**) *hCYP19A1*, (**c**,**d**) *hSRD5A1*, (**e**,**f**) *hSRD5A2* and (**g**,**h**) *hSRD5A3* in control subcutaneous adipose tissue (SAT, violet bars) and lipomatous tissue (LT, light blue bars) from MSL patients (**a**,**c**,**e**,**g**) and in respective SVF-derived cells (**b**,**d**,**f**,**h**). Expression levels are normalized to 18S rRNA content and are reported in arbitrary units (AUs). Data are presented as mean ± SEM; SAT: n = 9, LT: n = 14. Statistical analysis was performed using the Mann–Whitney test; ns: not significant. SAT: subcutaneous adipose tissue; LT: lipomatous tissue.

*hCYP19A1* encodes a member of the cytochrome P450 superfamily of enzymes also known as aromatase. Figure 1 shows its expression both in LT and control SAT of 8 MSL patients (panel a), as well as in the respective SVF cells (panel b). The expression of *hCYP19A1* in both tissues was low compared to placental tissue used as positive control. The mean value of *hCYP19A1*/*18S*, reported in arbitrary units (AUs), in 5 ng of cDNA of placenta was about 8.2 ± 2, while mean values obtained for control SAT and LT were respectively about 0.03 and 0.05 (ranging from 100 to 300 times lower). Moreover, *hCYP19A1* expression levels in LT were heterogeneous among different MSL patients, being higher, lower, or similar to those measured in the control SAT of the same patient (Figure S2).

*hSRD5A1* was expressed both in MSL-spared and MSL-affected tissues (Figure 1, panel c), as well as in the respective SVF cells (Figure 1, panel d). The expression of *hSRD5A1* in both tissues was similar to that found in fibroblast cells used as positive control. The *hSRD5A1* expression levels in LT were heterogeneous among different MSL patients, as reported in Figure S3. *hSRD5A2* was expressed at very low levels in LT and control SAT, as previously reported in the literature [24,25], and was undetectable in most analyzed samples (Figure 1, panels e and f, and Figure S4). *hSRD5A3* was expressed in both tissues, LT and control SAT (Figure 1, panel g), and in SVF cells (Figure 1, panel h) at a higher level than isoforms 1 and 2, as previously shown [24,25]. *hSRD5A3* expression levels were similar in whole tissue and SVF cells obtained from both control SAT and LT and variable among different MSL patients (Figure S5).

### 3.2. Quantification of Aromatase Activity

Different methods of tissue extraction have been employed to assess the specific aromatase activity, which is very low in SAT compared to placental tissue. Total tissue lysates and microsome preparations from whole tissue have been used in an attempt to concentrate the enzyme and to amplify its activity. Moreover, the incubation time was increased and co-factors (NADPH, G6P, G6PD) were added repeatedly during the reaction period. Nevertheless, specific aromatase activity, inhibited by different concentration of Letrozole and Exemestane, was detected in 50 µg, 50 ng and also 5 ng of microsomes obtained from placental tissue, while only very low aromatase activity (about 1000 times lower) was detected in 50 µg of microsomes obtained from LT of some MSL patients. No specific aromatase activity was detected in control SAT.

The quantification of aromatase activity in cell cultures was performed and the assay was set up using the JEG3 human placenta-derived cell line (50,000 or 100,000 cells/well) and 3T3-L1 mouse preadipocyte cell line (50,000 cells/well) seeded in their specific culture media, MEM 10%FBS and DMEM high-glucose 10%FBS, respectively. To demonstrate the specificity of the detected aromatase activity, cells were treated with both aromatase (Exemestane, Exe) and 5-α-reductase (Finasteride, Fin) inhibitors at the indicated concentrations. Under these conditions, specific aromatase activity was detected in JEG3 cells that were inhibited in a dose-dependent manner by Exemestane, while it was not influenced by the treatment with Finasteride. No specific aromatase activity was detected in 3T3-L1 mouse preadipocytes (Figure S6).

**Figure 2:**
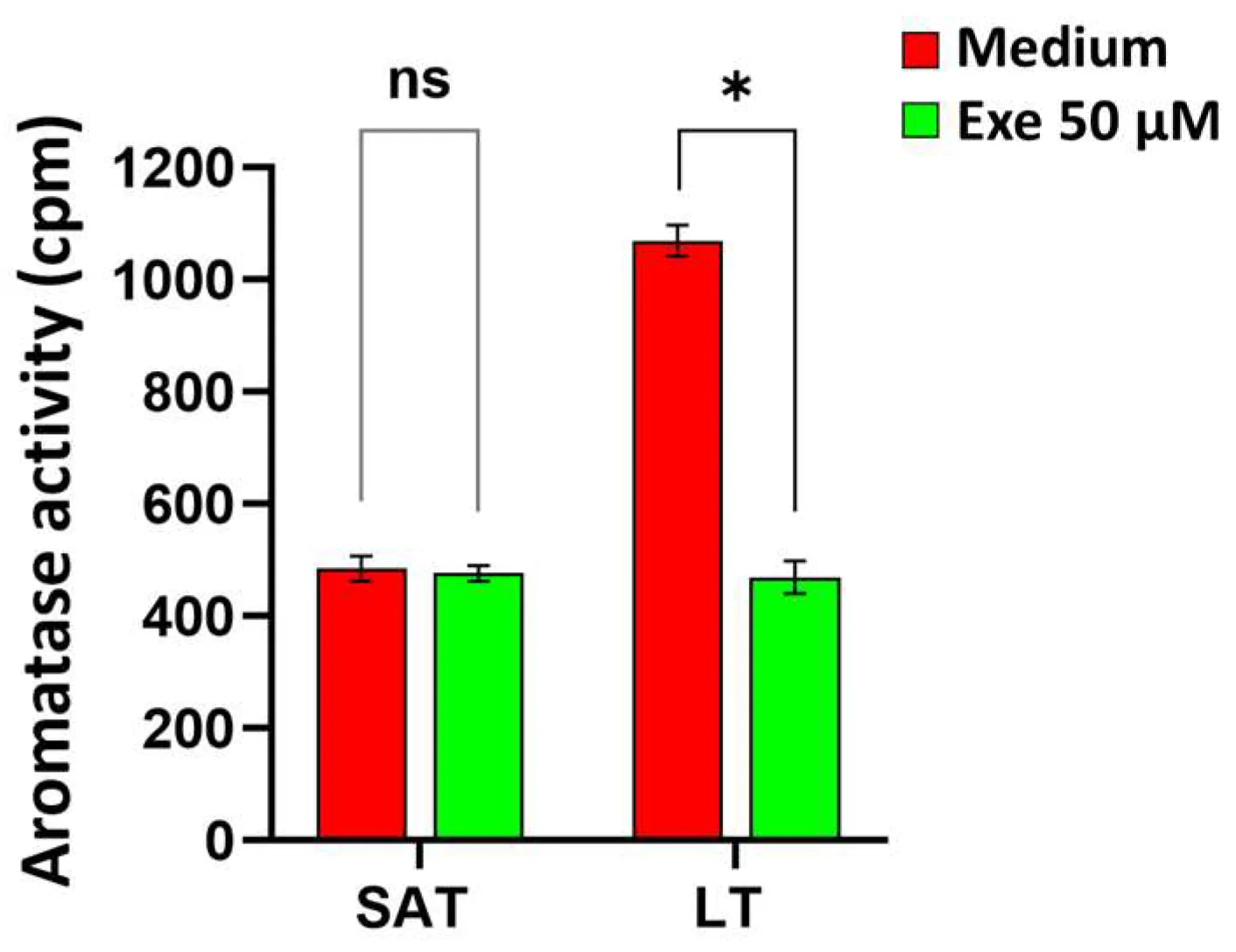
Aromatase activity in MSL-derived primary cultures. SVF cells obtained from control SAT and LT of patient ID 20922 cultured in αMEM 5% hPL were assayed for aromatase activity in standard medium (red bars) and in the presence of 50 µM Exemestane (green bars). Data are reported in cpm (count per minute) as mean of quadruplicates ± SEM and are representative of 2 independent experiments. Statistical analysis was performed by Mann–Whitney test for multiple comparisons. ns: not significant; *: *p* < 0.05.

The aromatase activity was measured with the same assay in SVF cells (50,000 cells/well) obtained from control SAT and LT of one patient cultured in αMEM 5%hPL. In the experiment reported in Figure 2, specific aromatase activity (inhibited by treatment with Exemestane) was detected only in LT-derived SVF cells. This aromatase activity was between 8 and 60 times lower than that quantified in independent experiments performed using JEG3 cells.

### 3.3. Cell Viability and Proliferation of Primary Cultures from MSL Patient in Presence of Aromatase and 5-α-Reductase Inhibitors

**Figure 3:**
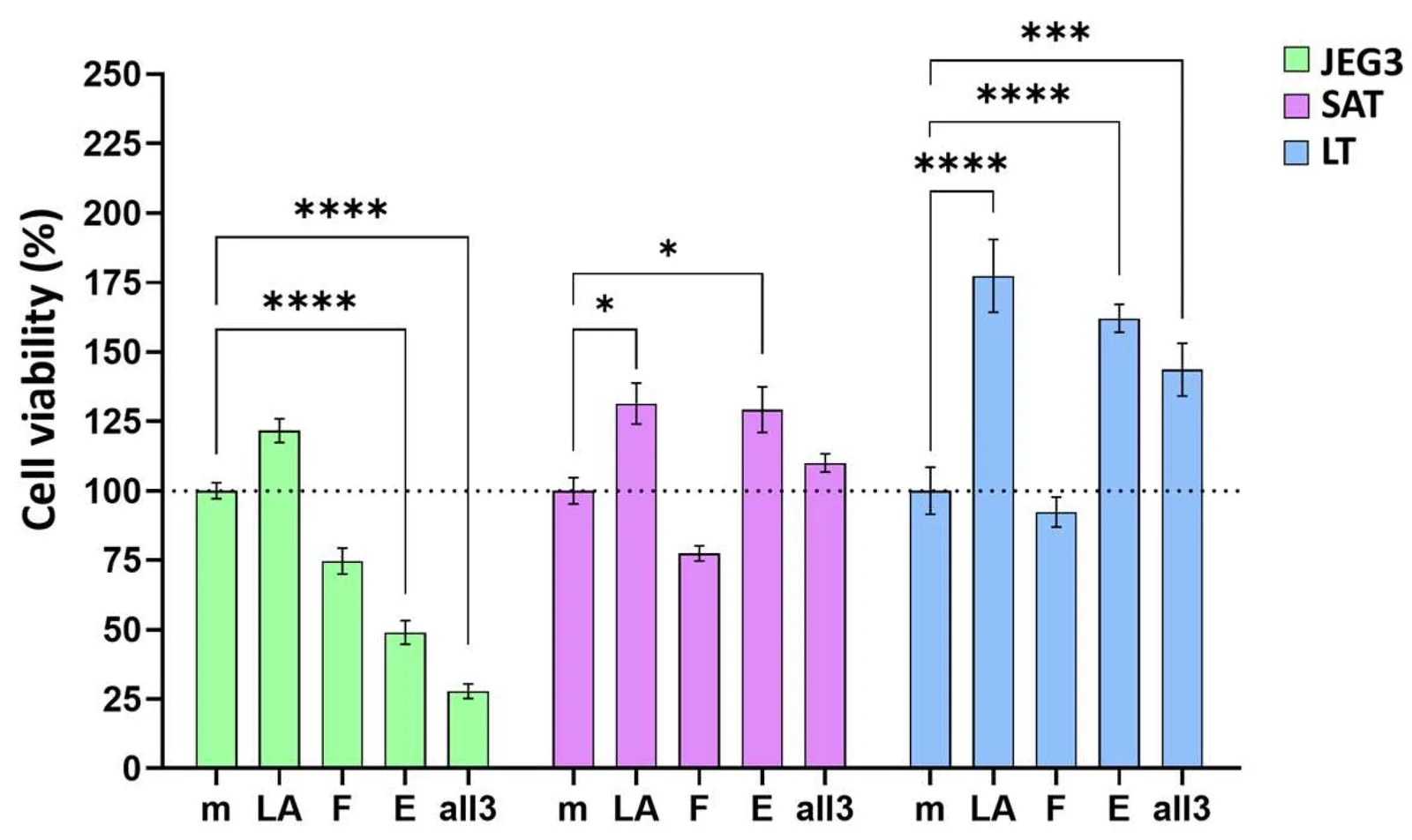
Cell viability assay of MSL-derived primary cultures in the presence of DL-α-Lipoic Acid, Finasteride and Exemestane. JEG3 cells (green bars) and SVF cells obtained from control SAT (violet bars) and LT (light blue bars) of patient ID 20922 were treated with DL-α-Lipoic Acid (LA, 250 µM), Finasteride (F, 50 µM), Exemestane (E, 50 µM) and all 3 compounds (all3) for 48 h in MEM 10%FBS and DMEM F12 10%FBS, respectively. Data are reported as mean of triplicates ± SEM and are representative of 2 independent experiments. Untreated cells (medium alone, m) were set to 100 for each cell type (as indicated by the dotted line) and data are shown as percentage (%) of cell viability. Statistical analysis was performed by ordinary one-way ANOVA followed by Dunnett’s post hoc test, comparing treated vs. untreated cells for each cell type; *: *p* < 0.05, ***: *p* < 0.001, ****: *p* < 0.0001.

JEG3 cells, cultured in MEM 10%FBS, and SVF cells obtained from control SAT and LT of one MSL patient (ID 20922) cultured in DMED F12 10%FBS, were treated with the different compounds, alone or in combination, at the indicated concentrations for 48 h. Figure 3 shows the cell viability expressed as the luminescence signal which correlates to the number of viable cells in the different experimental conditions. The untreated cells (medium alone, m) served as internal control and were set to 100 for each cell type. Cells treated with DL-α-Lipoic Acid (LA) displayed a slight increase in the cell viability signal while cells treated with Finasteride (F) displayed a similar (in LT) or slight decrease in the cell viability signal. JEG3 cells displayed a relevant reduction in cell viability upon treatment with Exemestane (E) and an even greater reduction with the combination of the three compounds (all3). On the contrary, the same treatments slightly increased the cell viability of SAT and especially that of LT-derived cells.

**Figure 4:**
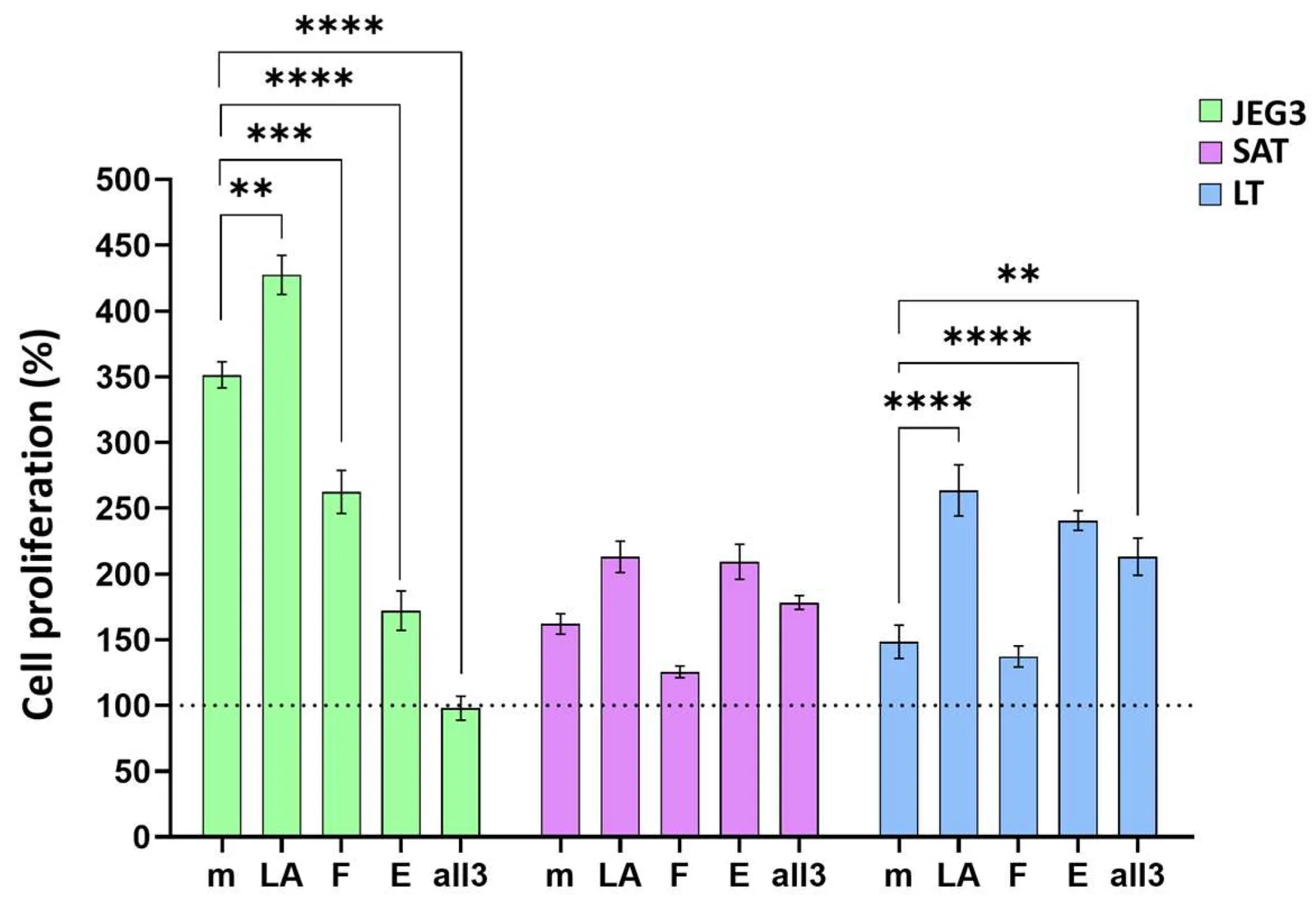
Cell proliferation analysis of MSL-derived primary cultures in the presence of DL-α-Lipoic Acid, Finasteride and Exemestane. JEG3 cells (green bars) and SVF cells obtained from control SAT (violet bars) and LT (light blue bars) of patient ID 20922 were treated with medium alone (m), DL-α-Lipoic Acid (LA) (250 µM), Finasteride (F) (50 µM), Exemestane (E) (50 µM) and all 3 compounds (all3) for 48 h in MEM 10%FBS and DMEM F12 10%FBS, respectively. Cell proliferation was calculated by dividing the luminescence signal obtained after 48 h of treatment by the luminescence signal obtained at time 0 (24 h after cell seeding) for each cell type. Data, representative of 2 independent experiments, are reported in percentage (%) as mean of triplicates ± SEM, setting the time 0 at 100% for each cell type (dotted line). Statistical analysis was performed by ordinary one-way ANOVA followed by Dunnett’s post hoc test, comparing treated vs. untreated cells for each cell type. **: *p* < 0.01, ***: *p* < 0.001, ****: *p* < 0.0001.

The results were also analyzed and represented as proliferation percentages, dividing the luminescent signal obtained after 48 h of each treatment by the basal luminescence signal measured at time 0 (the day after cell seeding). Figure 4 shows the result obtained for each condition setting the relative basal luminescence signal as 100% (dotted line). In untreated conditions (medium alone, m), JEG3 cells displayed a higher proliferation capacity (351%) than control SAT (162%) and LT (148%). JEG3 cells displayed a slight increase in cell proliferation in the presence of DL-α-Lipoic Acid (LA) and a progressive decrease in cell proliferation upon treatment with Finasteride (F), Exemestane (E) and the combination of the three compounds (all3). Moreover, among the treatments tested, LA, E and all3 slightly increased cell proliferation in both SAT- and LT-derived SVF cells, an effect that reached statistical significance specifically in LT-derived cells. In contrast, Finasteride (F) slightly decreased cell proliferation in both cell populations. Morphological observations under optical microscopy performed during the assay confirmed the results observed through luminescence signal reading.

### 3.4. Adipogenic Differentiation in the Presence of Aromatase and 5-α-Reductase Inhibitors

**Figure 5:**
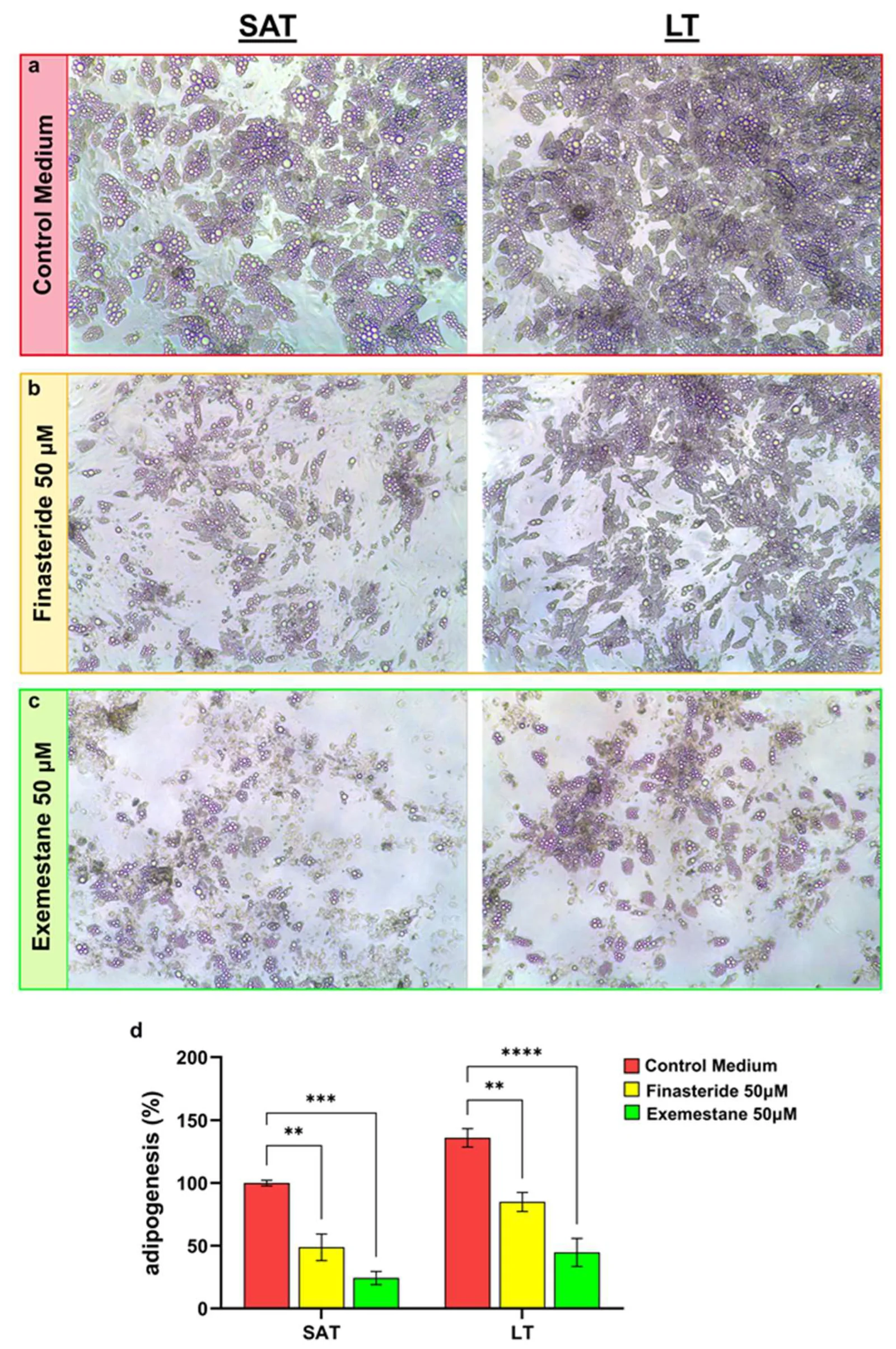
Adipogenic differentiation of MSL-derived SVF cells with chemical enzyme inhibitors. SVF cells obtained from control SAT and LT of MSL patient ID 20922 were differentiated for 12 days in (**a**) hMIR/MAD (control medium), (**b**) in the presence of Finasteride 50 µM or (**c**) Exemestane 50 µM. (**d**) Quantification of adipogenesis in percentage was estimated by phase contrast microscopy and reported as mean value ± SEM for cells differentiated in standard adipogenic conditions (control medium) (red bars), supplemented with Finasteride 50 µM (yellow bars) or Exemestane 50 µM (green bars). Experiments were performed twice analyzing at least 5 images per condition. Representative images acquired by optical microscopy at 10X magnification are shown. Statistical analysis was performed by ordinary two-way ANOVA followed by Tukey’s post hoc test for multiple comparisons; **: *p* < 0.01, ***: *p* < 0.0001, ****: *p* < 0.0001.

SVF cells obtained from control SAT and LT of MSL patient ID 20922 were cultured in DMED F12 10%FBS or in αMEM 5%hPL and induced to undergo adipogenic differentiation using a standard protocol for human primary cultures. In general, adipogenic differentiation experiments gave better results when SVF cells were seeded in DMEM F12 10%FBS rather than in αMEM 5%hPL, probably because under those conditions cells were homogenously distributed and reached the optimal degree of confluence. In all experiments, SVF cells obtained from LT displayed a higher adipogenic differentiation potential compared to those obtained from control SAT across all different culture conditions (Figure 5). When treated with DL-α-Lipoic Acid (LA) 250 μM alone or in combination with the other compounds (DL-α-LA + Finasteride + Exemestane) during the adipogenic differentiation, SVF cells appeared stressed, with many dead cells and many cells failing to proliferate or differentiate, except for a few isolated adipocytes in some wells and conditions (Figure S7). Conversely, the treatment with Finasteride (50 μM) alone reduced the adipogenic differentiation capacity of both LT- and control SAT-SVF cells compared to control medium (Figure 5, panels b and d). Moreover, the treatment with Exemestane (50 μM) under adipogenic conditions significantly reduced adipogenesis of both SVF cell populations (Figure 5, panel d) and the adipogenic process seemed to be slower, giving rise to fewer isolated and rounded adipocytes characterized by smaller lipid vacuoles compared to control medium (Figure 5, panel c). Figure 5 shows representative images (panels a–c) and relative quantification of adipogenesis (panel d) in one of the two experiments performed.

## 4. Discussion

This study investigates the potential role of sex hormone metabolism in the pathophysiology of MSL and explores a possible pharmacological approach to managing the disease.

The findings show the expression of aromatase (*CYP19A1*) and 5-α-reductase isoforms (*SRD5A1*, *SRD5A2*, and *SRD5A3*) in both MSL-affected (LT) and MSL-spared (control SAT) biopsies of MSL patients at different levels. Moreover, notably, the expression of these enzymes is similar in whole tissue compared with SVF cells, suggesting similar levels and activity in mature adipocytes and adipose precursor cells.

The expression of aromatase (*CYP19A1*) is approximately 100–300 times lower in LT compared with placental tissue. Studying and quantifying specific aromatase activity in AT is very challenging due to its very low levels. Based on previously established protocols, a highly sensitive and specific radioactive assay based on a tritiated water-release method was developed, using placental tissue and human JEG3 cells as positive controls, to quantify aromatase activity in tissue and cell culture samples. Thus, the low but detectable levels of aromatase expression and activity demonstrated in MSL-affected tissue support the hypothesis that local estrogen synthesis could contribute to adipogenesis and LT growth. Notably, the observed heterogeneity in *CYP19A1* expression across different MSL patients suggests that individual variability in local estrogen metabolism may play a role in disease progression and response to treatment. Moreover, the ability of Exemestane to inhibit aromatase activity in whole LT and LT-derived primary cultures, albeit at low levels, highlights the potential for targeting local estrogen synthesis as a potential therapeutic strategy.

Regarding the expression of 5-α-reductase isoforms, the data shown herein confirm previous findings demonstrating that the expression of isoform 3 is similar or slightly higher than isoform 1 and both isoforms 3 and 1 are more highly expressed than isoform 2 in LT as well as in control SAT (*SRD5A3* ≥ *SRD5A1* >> *SRD5A2*). Accordingly, these results indicate that 5-α-reductase isoforms 3 and 1 could potentially be involved in modulating intracellular androgen metabolism in LT. The variability in *SRD5A3* expression observed among different MSL patients further underlines the complexity of androgen metabolism in this notably rare condition.

Exposure to DL-α-Lipoic Acid alone increased cell viability and proliferation capacity in all tested cell cultures (JEG3 control cell line, LT and SAT-derived SVF cells), suggesting a potential role in enhancing cellular metabolic activity and survival pathways in precursor cells.

Interestingly, JEG3 cells, used as a control in the cellular experiments, displayed a significant reduction in cell viability and proliferation upon treatment with both 5-α-reductase and aromatase inhibitors alone or in combination. However, SVF cells obtained from MSL-affected and MSL-spared tissue samples displayed only a slight increase in cell viability and proliferation upon treatment with the aromatase inhibitor, which reached statistical significance for LT-derived precursor cells. These data suggest that placental choriocarcinoma-derived tumor cells (JEG3) are probably strictly dependent on hormonal stimulation for their survival, while LT- (in particular) and SAT-derived precursor cells could be regulated by sex hormones in their metabolism, proliferation and differentiation potential. Overall, SVF cells obtained from LT displayed a slight increase in the proliferation capacity compared to those obtained from the paired control SAT of the same patient. Similar observations were made during routine subculture passages for expansion, confirming our previous data [11].

Furthermore, it was demonstrated that LT-derived SVF cells exhibited a higher capacity to differentiate into mature adipocytes under all adipogenic conditions and treatments tested in comparison with control SAT-derived cell cultures, as previously reported in other MSL patients [11]. Although the treatment with aromatase and 5-α-reductase enzyme inhibitors reduced the adipogenesis in both LT and control SAT cultures, in LT-derived cells it was able to counteract a greater intrinsic adipogenic potential. Moreover, the expression of these enzymes varied in affected and non-affected tissues depending on the patient considered. These observations support the hypothesis that targeting local sex hormone metabolism could modulate LT expansion in MSL in a personalized manner. Moreover, the greater effect of Exemestane treatment on the reduction in adipogenesis suggests that aromatase inhibition may be particularly effective in controlling LT growth. However, although the mechanisms by which this enzymatic inhibition negatively impacts adipogenesis have not been fully elucidated, the present results obtained by evaluating cell proliferation suggest that it is not through the inhibition of precursor mitotic clonal expansion [31].

Lastly, MSL is reportedly associated with chronic alcohol abuse. These patients often display a disrupted hypothalamic–pituitary–gonadal axis with reduced systemic testosterone levels [32]. Alcohol can also modulate aromatase activity [33] and may shift the local estrogen/androgen balance in AT and particularly in LT. The endocrine consequences of chronic alcohol intake could therefore create a permissive environment for dysfunctional adipogenesis through local steroidogenesis and impaired hormonal feedback. Nevertheless, non-hormone-related mechanisms may also influence LT accumulation in MSL, such as non-steroid-mediated metabolic pathways and microenvironmental effects. In this regard, the mTOR signaling pathway has been proposed as an additional non-hormonal mechanism that contributes to MSL, as its inhibition by rapamycin was shown to modulate the aberrant proliferation and differentiation behavior observed for LT-derived ASCs [34]. Moreover, targeting the AT microenvironment through modulation of its stromal compartment, local hormonal milieu and extracellular matrix remodeling may therefore represent a promising therapeutic strategy to interfere with aberrant adipogenic differentiation and tissue architecture in MSL. Notably, this approach mirrors emerging paradigms in oncology, where manipulation of the tumor microenvironment and surrounding matrix has been shown to alter not only cancer cell behavior but also the penetration and efficacy of therapeutic agents [35]. However, putative targeting of these mechanisms and related molecular pathways requires further investigation.

Several limitations should be considered when interpreting our findings. First, although this study represents one of the largest single-center cohorts of patients with MSL reported to date, the overall sample size remains limited due to the ultra-rare nature of the disease. Consequently, the statistical power for detecting subgroup differences may be reduced. Furthermore, this is an in vitro study and therefore only preclinical results are reported. Moreover, as it is particularly difficult to obtain LT biopsies suitable for in vitro cell culture experiments, gene expression analyses could be performed in multiple MSL patients, whereas functional studies, including cell viability, proliferation and adipogenic differentiation assays, could only be carried out in a single MSL patient (ID 20922). In particular, it must be noted that the effects of the tested compounds on adipogenic markers were not determined, as insufficient material was available to perform mRNA or protein expression analyses. Nevertheless, we are reasonably confident that morphological observations allow us to clearly identify mature adipocyte cells in human cultures. Another limitation was the observed variability in aromatase and 5-α-reductase expression amongst LT samples, making it difficult to draw general conclusions regarding the role of sex hormones across all MSL patients. In this regard, further experiments should be planned in a larger number of MSL patients to compare the transcriptome and proteome of LT and SAT in both whole tissue and SVF cells (containing adipogenic precursors) to identify cell populations and signaling pathways involved in the increased and dysfunctional adipogenesis observed in LT.

It is worth noting that MSL is a highly variable disorder with a pathogenesis that is still unclear and limited current treatment approaches [36,37]. The results presented herein are only preliminary and were obtained from a limited number of patients, yet they maintain the relevance of the study due to the orphan disease status of MSL. Our results provide the basis for further pharmacological studies targeting hormonal pathways as a potential new treatment for MSL.

## 5. Conclusions

The results of this preclinical study demonstrated that both aromatase and 5-α-reductase genes were expressed in LT of MSL patients, and low but specific aromatase activity was detected in LT-derived SVF cells.

Treatment of primary precursor cultures (obtained from LT) with aromatase inhibitors, alone or in combination, increased cell viability and proliferation capacity, but reduced their adipogenic potential. These results could provide a biological basis for the use of these chemical inhibitors as a potential new treatment for MSL. However, future multi-center and international collaborative studies with larger cohorts are needed to validate and extend these preliminary findings.

## Figures and Tables

**Table 1:** Anthropometric and clinical characteristics of MSL patients at the time of surgery. Abbreviations: ID: identification number of the patients; #1 and #2: number of the different interventions; MSL: multiple symmetric lipomatosis; BMI: body mass index; LT: lipomatous tissue; SAT: subcutaneous adipose tissue; T2DM: Type 2 Diabetes Mellitus; alcohol: habit of alcohol consumption two years before enrollment into the study.

ID Patient	Gender	Age	MSL Type	LT Site	SAT	BMI	T2DM	Alcohol
2884	M	58	1	Cervical	Abdominal	22.5	No	Yes
2891#1	M	62	1	Neck	Abdominal	26.6	No	Yes
2891#2	M	63	1	Cervical and neck	Abdominal	26.5	No	Yes
3002#1	M	64	1	Neck and upper arm	Lumbar region	29.7	No	Yes
3030	M	49	1	Upper arm	Abdominal	29.4	Yes	No
3662	F	64	1	Upper arm	Abdominal	34.3	Yes	Yes
4450	F	70	2	Upper arm	Abdominal	36.7	No	Yes
5091	M	70	2	Neck and upper arm	Abdominal	28.3	No	Yes
5937	F	63	1	Upper arm	Abdominal	33.8	Yes	Yes

**Table 2:** Real-Time PCR conditions. Primer sequences and concentrations, annealing temperatures and amplicon sizes are outlined for each mRNA quantified by Real-Time PCR. *rRNA18S*: 18S ribosomal RNA; *hCYP19A1*: human cytochrome P450 family 19 subfamily A member 1; *hSRD5A1*: human steroid 5-alpha-reductase 1; *hSRD5A2*: human steroid 5-alpha-reductase 2; *hSRD5A3*: human steroid 5-alpha-reductase 3.

Gene	Forward (5′-3′)	Reverse (5′-3′)	Primer F/R (nM)	Annealing (°C)	Amplicon Size (bp)
*rRNA18S*	CGGCTACCACATCCAAGGA	GCTGGAATTACCGCGGCT	100/100	60	186
*hCYP19A1*	TGCAAAGCACCCTAATGTTG	TGGTACCGCATGCTCTCATA	300/300	60	135
*hSRD5A1*	TACCAAGGGGAGGCTTATTT	GGTACCACTCATGATGCTCTT	300/300	60	168
*hSRD5A2*	TGCCTACATTACTTCCACAGGACA	ACCCATCAGGGTATTCAGCACAGT	300/300	60	157
*hSRD5A3*	TCCTTGTTGGCCTAACTGTGCT	ACCACTCCTGCTTTATTTTTCCTGA	300/300	60	193

## Data Availability

The raw data supporting the conclusions of this article will be made available by the authors on request.

## Supplementary Materials

The following supporting information can be downloaded at: https://www.mdpi.com/article/10.3390/biomedicines14081706/s1.

## Abbreviations

The following abbreviations are used in this manuscript:

hCYP19A1	Human Cytochrome P450 Family 19 Subfamily A Member 1 (Aromatase)
hSRD5A1	Human Steroid 5-α-Reductase 1
hSRD5A2	Human Steroid 5-α-Reductase 2
hSRD5A3	Human Steroid 5-α-Reductase 3
SRD5A	Steroid 5-α-Reductase
RNA18S	18S Ribosomal RNA
MSL	Multiple Symmetric Lipomatosis
LT	Lipomatous Tissue
SAT	Subcutaneous Adipose Tissue
AT	Adipose Tissue
SVF	Stromal Vascular Fraction
BAT	Brown Adipose Tissue
UCP1	Mitochondrial Uncoupling Protein 1
iNOS	Inducible Nitric Oxide Synthase
PGC-1	Peroxisome Proliferator-Activated Receptor γ Co-activator 1
PKB	AKT/Protein Kinase B
CK2	Protein Kinase CK2
ERK1/2	Extracellular Signal-Regulated Kinase 1/2
NADPH	Nicotinamide Adenine Dinucleotide Phosphate
G6P	Glucose-6-Phosphate
G6PD	Glucose-6-Phosphate Dehydrogenase
ASCs	Adipose Stem Cells
mTOR	Mechanistic Target Of Rapamycin
DL-α-LA	DL-α-Lipoic Acid
E	Exemestane
F	Finasteride
BMI	Body Mass Index
T2DM	Type 2 Diabetes Mellitus
hMAD	Human Medium for ADipogenesis
hMIR	Human Medium for ADipogenesis with IBMX and Rosiglitazone
hPL	Human Platelet Lysate
FBS	Fetal Bovine Serum
DMSO	Dimethyl Sulfoxide
IBMX	3-Isobutyl-1-Methylxanthine
T3	3,3′,5-Triiodo-L-Thyronine
PBS	Phosphate-Buffered Saline
MEM	Minimum Essential Medium
DMEM	Dulbecco’s Modified Eagle Medium
