# Supplementary material for: Targeting Aromatase and 5-α-Reductase to Limit Dysfunctional Adipogenesis in Multiple Symmetric Lipomatosis
Source: Biomedicines. 2026 Jul 29;14(8):1706. doi: 10.3390/biomedicines14081706 (PMC13509339; doi:10.3390/biomedicines14081706)
Supplement: Supplementary file 1 [file biomedicines-14-01706-s001.zip › biomedicines-4411256-supplementary.pdf]

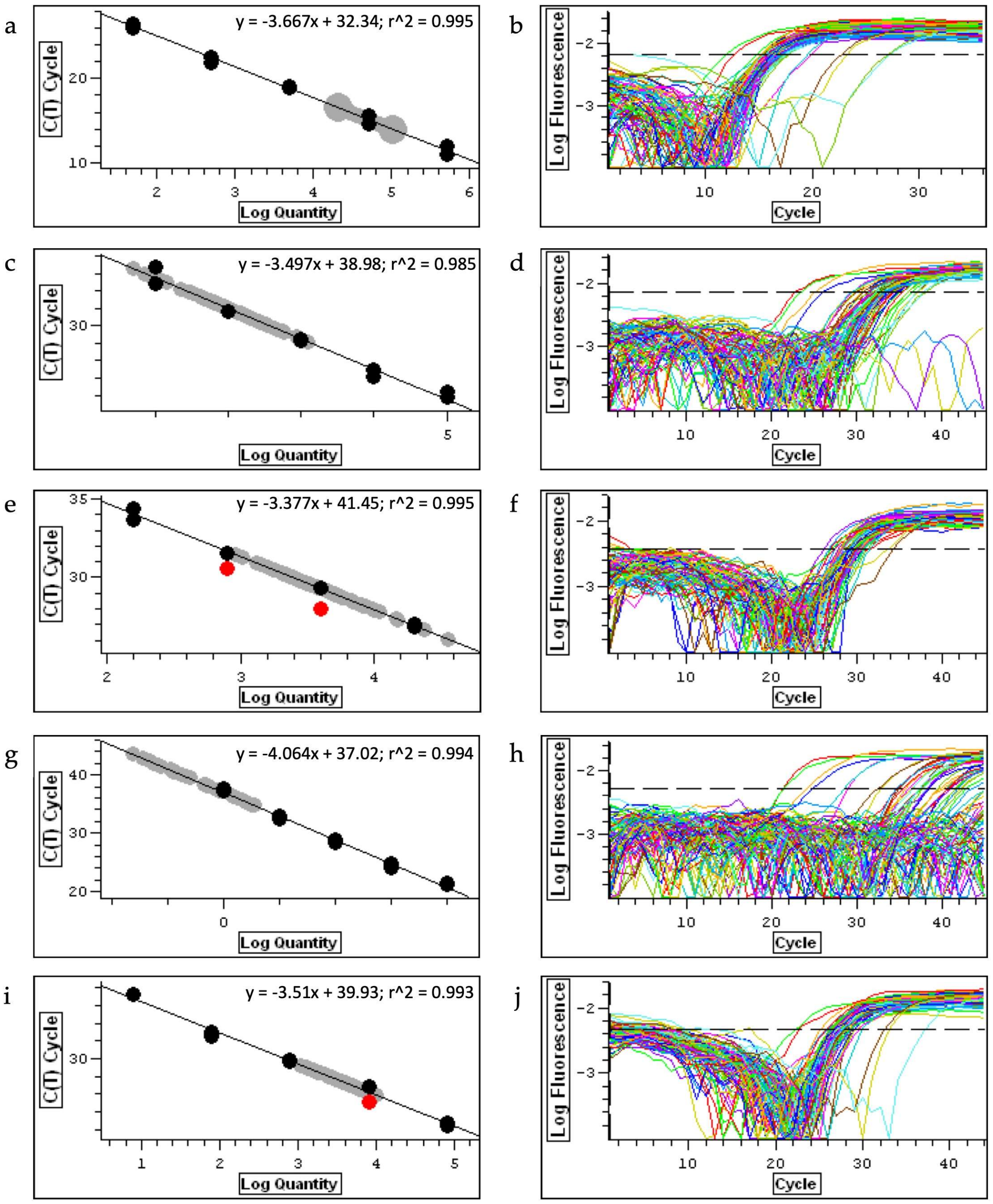

Figure S1. Standard curve (a,c,e,g,i) and amplification plots (b,d,f,h,j) of Real Time PCR of *rRNA18S* (a,b), *hCYP19A1* (c,d), *hSRD5A1* (e,f), *hSRD5A2* (g.h) and *hSRD5A3* (i,j) performed in biopsies of 8 MSL patients. The black circles represent cDNA dilution series 1:10 (a, c, e, i) or 1:5 (g) and red circles are the samples excluded. The grey circles are the samples analysed in duplicates. *RNA18S*: 18S ribosomal RNA; *hCYP19A1*: human cytochrome P450 family 19 subfamily A member 1; *hSRD5A1*: human steroid 5-alpha-reductase 1; *hSRD5A2*: human steroid 5-alpha-reductase 2; *hSRD5A3*: human steroid 5-alpha-reductase 3.

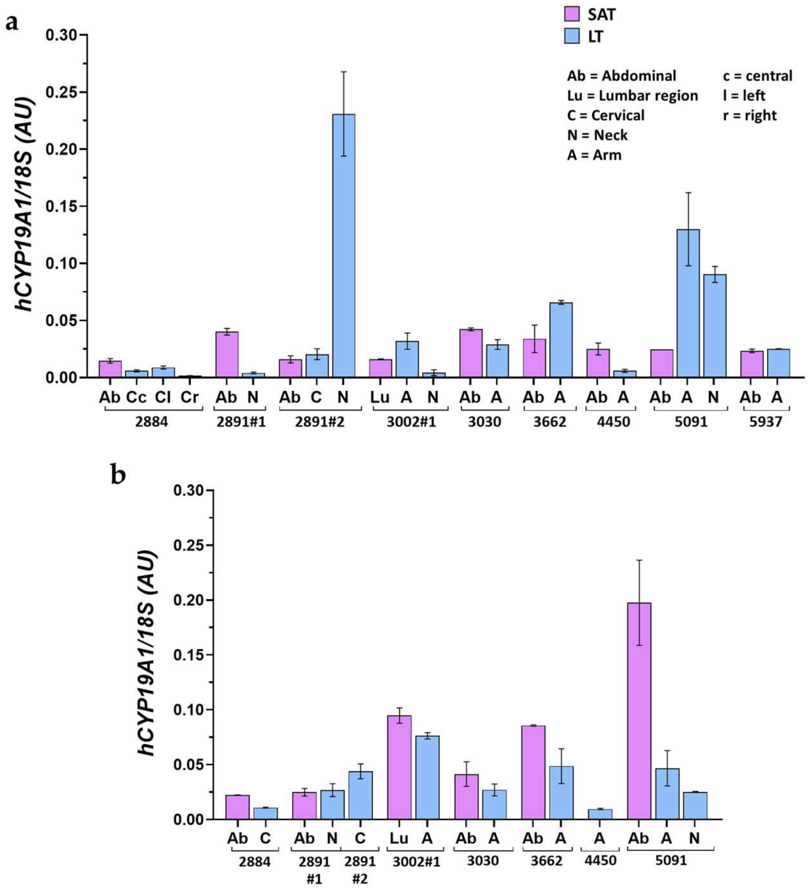

Figure S2. *hCYP19A1* expression profile in whole lipomatous tissue (panel **a**) and in SVF cells (panel **b**) obtained from 8 MSL patients. (**a**) *hCYP19A1* mRNA levels in whole LT (light blue bars) and control SAT (violet bars) and (**b**) in the corresponding LT-derived (light blue bars) and control SAT-derived (violet bars) SVF cells, normalized to *18S* rRNA content, are reported as mean of duplicates ± SEM. SAT: n = 9; LT: n = 14. The patient ID and the anatomical localization are indicated for each sample (see also Table 1). SAT: Subcutaneous Adipose Tissue; LT: Lipomatous Tissue; Ab: abdominal; Lu: Lumbar region; C: cervical; N: neck; A: arm; c: central; l: left; r: right.

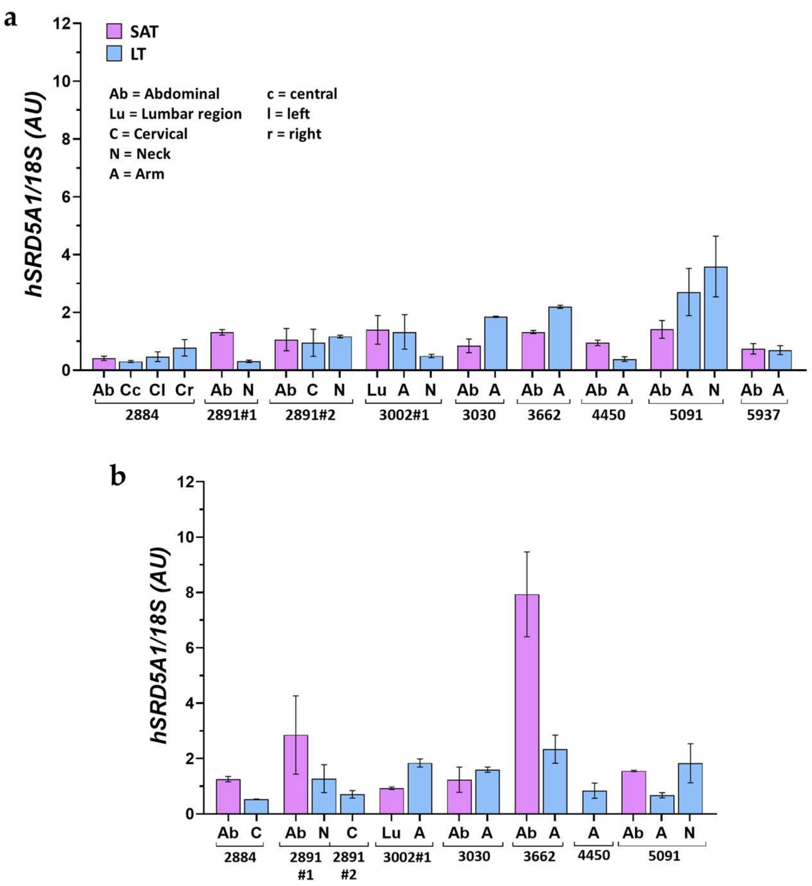

Figure S3. *hSRD5A1* expression profile in whole lipomatous tissue (panel **a**) and in SVF cells (panel **b**) obtained from 8 MSL patients. (**a**) *hSRD5A1* mRNA levels in whole LT (light blue bars) and control SAT (violet bars) and (**b**) in the corresponding LT-derived (light blue bars) and control SAT-derived (violet bars) SVF cells, normalized to *18S* rRNA content, are reported as mean of duplicates ± SEM. SAT: n = 9; LT: n = 14. The patient ID and the anatomical localization are indicated for each sample (see also Table 1). SAT: Subcutaneous Adipose Tissue; LT: Lipomatous Tissue; Ab: abdominal; Lu: Lumbar region; C: cervical; N: neck; A: arm; c: central; l: left; r: right.

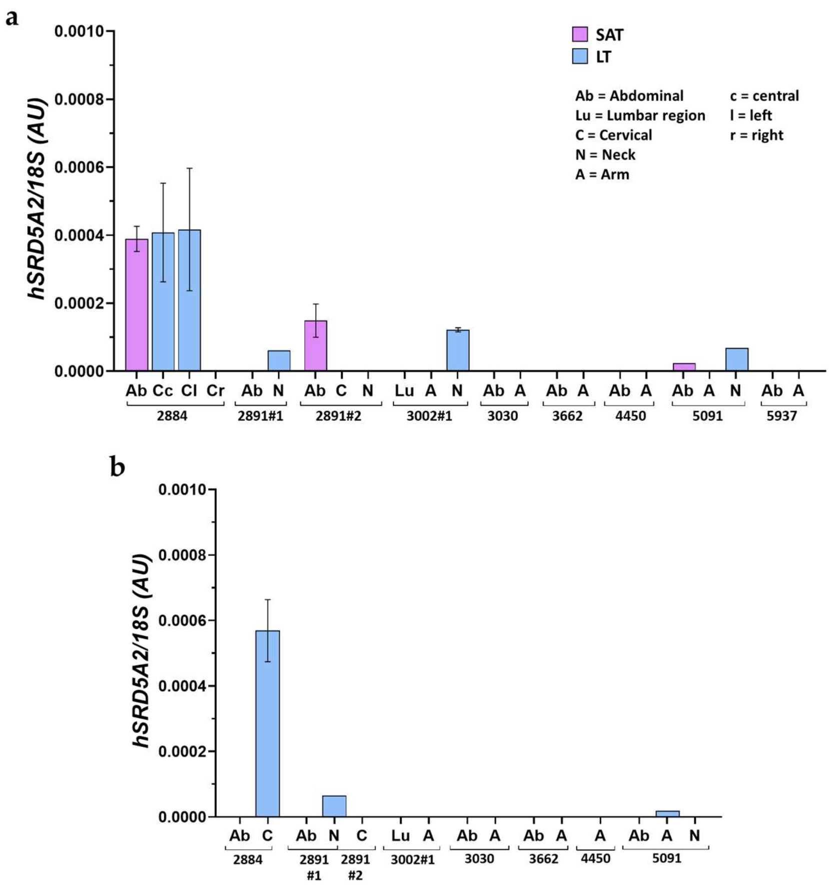

Figure S4. *hSRD5A2* expression profile in whole lipomatous tissue (panel **a**) and in SVF cells (panel **b**) obtained from 8 MSL patients. (**a**) *hSRD5A2* mRNA levels in whole LT (light blue bars) and control SAT (violet bars) and (**b**) in the corresponding LT-derived (light blue bars) and control SAT-derived (violet bars) SVF cells, normalized to *18S* rRNA content, are reported as mean of duplicates ± SEM. SAT: n = 9; LT: n = 14. For each sample, the patient ID and the anatomical localization are indicated (see also Table 1). SAT: Subcutaneous Adipose Tissue; LT: Lipomatous Tissue; Ab: abdominal; Lu: Lumbar region; C: cervical; N: neck; A: arm; c: central; l: left; r: right.

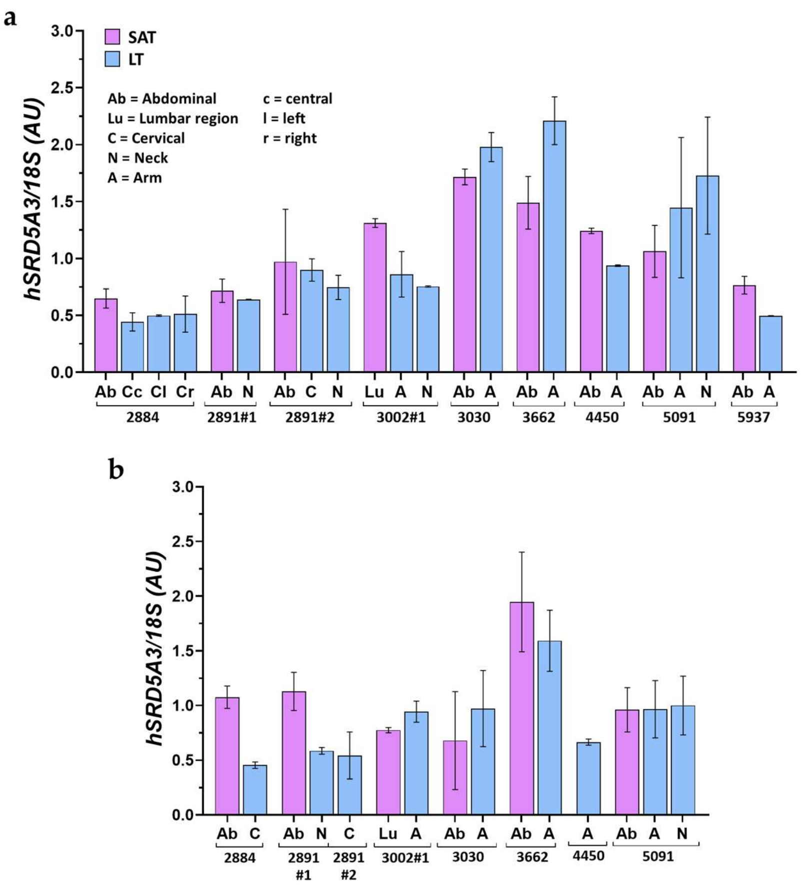

Figure S5. *hSRD5A3* expression profile in whole lipomatous tissue (panel **a**) and in SVF cells (panel **b**) obtained from 8 MSL patients. (**a**) *hSRD5A3* mRNA levels in whole LT (light blue bars) and control SAT (violet bars) and (**b**) in the corresponding LT-derived (light blue bars) or control SAT-derived (violet bars) SVF cells, normalized to *18S* rRNA content, are reported as mean of duplicates ± SEM. SAT: n = 9; LT: n = 14. For each sample, the patient ID and the anatomical localization are indicated (see also Table 1). SAT: Subcutaneous Adipose Tissue; LT: Lipomatous Tissue; Ab: abdominal; Lu: Lumbar region; C: cervical; N: neck; A: arm; c: central; l: left; r: right.

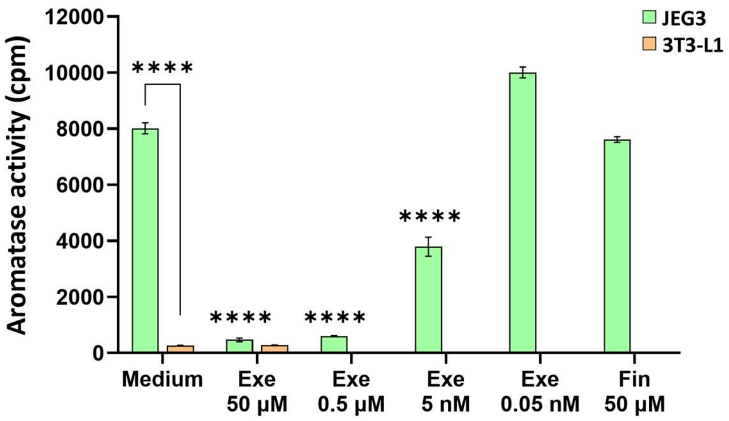

Figure S6. Setting-up of aromatase activity assay in cell cultures. JEG3 (green bars) and 3T3-L1 (orange bars) cells were untreated (medium) or treated with Exemestane (Exe) or with Finasteride (Fin), at the different concentrations indicated in a dose-dependent manner. The aromatase activity was measured

by the tritiated water-release assay, as described in the Materials and Methods section. Data are reported in cpm (count per minute) as mean of quadruplicates ± SEM and are representative of 3 independent experiments. Statistical analysis was performed by two-way ANOVA followed by Tukey's post-hoc test comparing untreated JEG3 vs untreated 3T3-L1 cells and treated vs untreated JEG3 cells; ****: $p < 0.0001$.

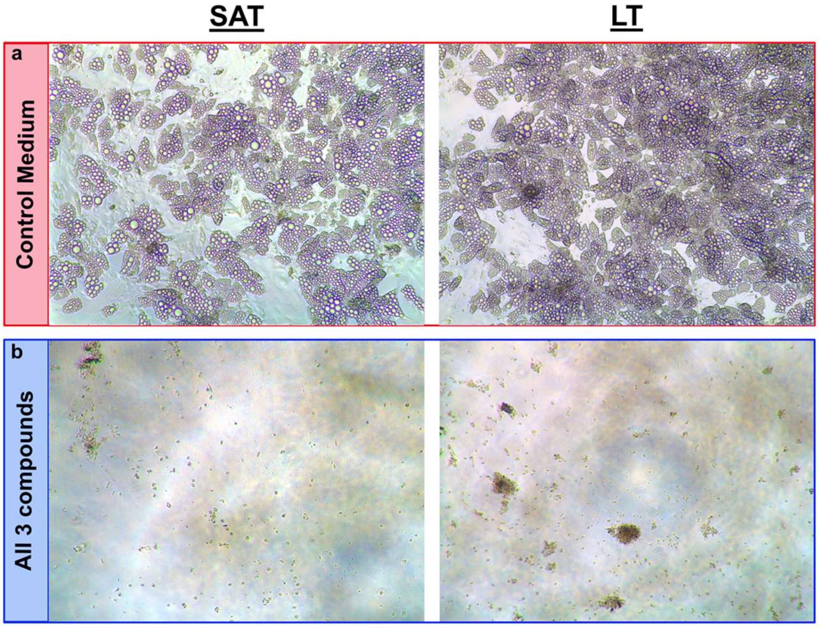

Figure S7. Adipogenic differentiation of MSL-derived SVF cells during treatment with a combination of all 3 chemical compounds. SVF cells obtained from control SAT and LT of MSL patient ID 20922 were differentiated for 12 days in (**a**) hMIR/MAD (control medium) or (**b**) in the presence of a combination of DL-α-LA (250 μM), Finasteride (50 μM) and Exemestane (50 μM) (all3). Representative images acquired by optical microscopy at 10X magnification are shown.
